# Protective Effects of Panax ginseng Extract on Endothelin-1- and Isoproterenol-Induced Cardiac Hypertrophy via Maintenance of Mitochondrial Function and Calcium-Reactive Oxygen Species (ROS) Homeostasis

**DOI:** 10.7759/cureus.99981

**Published:** 2025-12-23

**Authors:** Hideaki Tagashira, Fumiha Abe, Midori Yoshizaki, Tomohiro Numata

**Affiliations:** 1 Department of Integrative Physiology, Graduate School of Medicine, Akita University, Akita, JPN; 2 School of Medicine, Akita University, Akita, JPN

**Keywords:** cardiac hypertrophy, cardioprotection, mitochondrial preservation, oxidative stress, panax ginseng

## Abstract

Background: Mitochondrial dysfunction and oxidative stress are central to the development of cardiac hypertrophy and heart failure. *Panax ginseng* (PG), a principal herb used in Kampo medicine, has been reported to exert cardioprotective effects; however, its intracellular actions under endothelin-1 (ET-1)- and β-adrenergic stress remain incompletely defined.

Purpose: To determine whether a standardized PG extract attenuates cardiac hypertrophy and whether its effects are associated with mitochondrial function and Ca^2+^-reactive oxygen species (ROS) homeostasis in complementary cellular and in vivo stress models.

Methods: Neonatal rat ventricular myocytes (NRVMs) were exposed to ET-1 to induce hypertrophy and mitochondrial fragmentation. Mitochondrial morphology, intracellular Ca²⁺, ROS, and adenosine triphosphate (ATP) levels were quantified by fluorescence imaging and biochemical assays. In mice, isoproterenol (ISO) was administered for 14 days to induce cardiac stress; PG was given orally. Cardiac structure and function were evaluated by histology and echocardiography. Expression of mitochondrial fusion/fission markers was analyzed. All experiments used predefined exclusion criteria and blinded analyses.

Results: PG was associated with reduced ET-1-induced hypertrophy in a concentration-dependent manner (IC_50_ = 7.5 µg/mL), was associated with reduced mitochondrial fragmentation and loss of membrane potential, preserved ATP levels, and mitigated increases in intracellular Ca^2+^ and ROS in NRVMs. In ISO-treated mice, oral PG (50 mg/kg/day for 14 days) improved systolic function, limited hypertrophic remodeling, and reduced interstitial fibrosis.

Conclusion: PG exhibits pharmacological cardioprotection associated with modulation of mitochondrial dynamics and attenuation of cellular stress responses. These findings support further investigation of PG as a mitochondria-engaging natural product with the potential to mitigate pathological cardiac remodeling and heart failure progression.

## Introduction

Heart failure (HF) is a leading cause of morbidity and mortality worldwide, often representing the final common stage of diverse cardiovascular diseases [1,2]. A hallmark of HF progression is pathological cardiac hypertrophy, which initially serves as a compensatory adaptation but ultimately leads to maladaptive remodeling and contractile dysfunction. Mounting evidence suggests that mitochondrial dysfunction, encompassing impaired bioenergetics, redox imbalance, and Ca^2+^ handling defects, is at the core of this transition [3-6]. Beyond an “energy-starvation” model, mitochondria contribute to disease through vicious cycles that involve metabolic bottlenecks, reactive oxygen species (ROS)-induced ROS generation, protein modification, and disturbed mitochondrial quality control (fusion-fission dynamics and mitophagy) [7-9]. While guideline-directed medical therapy, comprising β-blockers, angiotensin-converting enzyme (ACE) inhibitors, angiotensin receptor blockers (ARBs), and angiotensin receptor-neprilysin inhibitors, improves symptoms and survival through neurohumoral modulation, these agents only indirectly address organellar failure, leaving subcellular targets underexploited [7,10,11]. This gap has catalyzed interest in mitochondria-focused strategies, including modulation of mitochondrial dynamics/mitophagy and detoxification of oxidative/aldehydic stress, which show promise across various cardiac stress contexts, such as hypertrophy and ischemia-reperfusion injury [8,12,13].

Natural products have long been recognized as important sources of pharmacologically active agents. *Panax ginseng* (PG), a key constituent of the Japanese Kampo Moku-boi-to, has been traditionally prescribed for patients with edema, dyspnea, and frailty associated with HF. Its principal actives, ginsenosides, exhibit broad pharmacology relevant to heart disease, including antioxidation, anti-inflammation, vasomotor regulation, ion-channel and signaling modulation, lipid profile improvement, blood-pressure adjustment, enhancement of cardiac function, and reduced platelet adhesion; moreover, contemporary work increasingly dissects single ginsenosides to define mechanisms [14]. As one exemplar, ginsenoside Rg3 attenuates pathological remodeling in vitro and in vivo, suppressing Ang II-evoked hypertrophy/fibrosis and mitigating transverse aortic constriction (TAC)-induced remodeling, by repressing NLRP3 inflammasome activation and oxidative stress via SIRT1/NF-κB signaling [15]. However, the effects of a chemically characterized PG extract on mitochondrial integrity and associated stress pathways under endothelin-1 (ET-1)-driven cardiomyocyte stress and in vivo β-adrenergic injury remain unclear. To explicitly address this gap, we investigated the pharmacological cardioprotective effects of PG in both in vitro and in vivo models.

Against this backdrop, mitochondria-focused interventions are advancing from concept to translation. Recent overviews highlight a diversified pipeline, from dietary supplements and small molecules that modulate redox and bioenergetics to emerging approaches such as mitochondrial component/organelle transplantation, reflecting the centrality of organellar targets across diseases, including cardiovascular pathology [16]. Notably, non-pharmacological strategies also remodel cardiac mitochondrial health: systematic analyses indicate that exercise training improves respiration and mitochondrial quality control (biogenesis, dynamics, mitophagy) in ischemic heart disease models [17], and mechanistic work in post-myocardial infarction HF shows that exercise restores autophagic flux, enhances mitochondrial oxidative capacity, and improves cardiac function [18]. Together, these developments underscore that repairing mitochondrial quality-control networks is a viable therapeutic axis, providing a rationale to evaluate mitochondria-preserving natural products such as PG within rigorously controlled cellular and in vivo cardiac stress models.

To address these knowledge gaps, the present study was designed with three clearly defined objectives: (i) To determine whether PG attenuates ET-1-induced hypertrophic remodeling in neonatal rat ventricular myocytes (NRVMs) and to evaluate associated changes in mitochondrial morphology, Ca^2+^ handling, ROS accumulation, and adenosine triphosphate (ATP) levels; (ii) To assess whether orally administered PG mitigates cardiac dysfunction and structural remodeling in vivo using a mouse model of isoproterenol (ISO)-induced β-adrenergic stress; (iii) To explore whether the cellular actions of PG are associated with aspects of mitochondrial homeostasis, assessed via transcriptional profiling of fusion-fission regulators (Opa1, Mfn1, Mfn2, Drp1, Fis1, Mtfp1) and sensitivity testing with orthogonal pharmacological probes (MYLS22 as an Opa1 inhibitor; Mdivi-1 as a Drp1 inhibitor), without inferring direct molecular causation.

By integrating complementary cellular and animal models, this study aims to clarify the pharmacological role of PG as a mitochondria-engaging natural product and to define its potential relevance in mitigating pathological cardiac remodeling and the progression of HF.

## Materials and methods

Reagents and herbal preparations

The Japanese Kampo medicine, Moku-boi-to (MBT; TJ-36) and its constituent crude drugs were obtained as follows: MBT, *Panax ginseng* C.A. Meyer (PG), *Sinomenium acutum* stem (*Sinomeni Caulis et Rhizoma*; SA), Cinnamomi cortex (CC), and Gypsum Fibrosum (GP). MBT was purchased from Tsumura & Co. (Tokyo, Japan) as the intermediate product of "TSUMURA Moku-boi-to Extract Granules for Ethical Use" as shown on their website [19,20], and prepared as a stock solution at 250 mg/mL in dimethyl sulfoxide (DMSO), followed by dilution to the desired working concentrations. PG, SA, CC, and GP were obtained from Nakaya Hikojyuro Pharmacy (Ishikawa, Japan), a licensed pharmaceutical manufacturer that employs nationally licensed pharmacists and holds the required manufacturing/marketing authorizations under Japanese law, operating in compliance with Good Manufacturing Practice (GMP) and the Japanese Pharmacopoeia (JP) standards for herbal medicines. For experimental use, each crude drug powder was extracted with 100% DMSO at room temperature with gentle agitation, and the supernatant was collected and used as the dried extract. Extracts were dissolved in DMSO at a concentration of 50 mg/mL, while GP was suspended at a concentration of 100 mg/mL. Extracts were stored as aliquots at -20°C and freshly diluted before use. To ensure pharmacological reproducibility, MBT and PG were obtained as preparations manufactured in compliance with GMP standards defined by the Ministry of Health, Labour, and Welfare of Japan and the JP [21,22]. These preparations met rigorous quality control requirements exceeding the minimum standards. Batch numbers and quality analyses were recorded to guarantee consistency throughout the experiments. Furthermore, PG was chemically characterized by high-performance liquid chromatography (HPLC) fingerprinting to confirm the presence of major ginsenosides (e.g., Rg1, Rb1, Rc, Rd). ISO, carboxymethyl cellulose (CMC), and additional reagents, including Fluo-4 AM, H_2_DCFDA, and MitoTracker Red CMXRos (MitoTracker), were obtained as previously described [23]. ET-1 was purchased from Peptide Institute Inc. (Osaka, Japan), Mdivi-1 from Sigma-Aldrich (MO, USA), and MYLS22 from Selleck Chemicals (TX, USA).

Animals and in vivo treatment protocol

Male C57BL/6J mice (10-12 weeks old) were housed under controlled environmental conditions (25 ± 1°C, 12-h light/dark cycle, ad libitum access to food and water). All experimental procedures complied with the institutional guidelines and were approved by the Animal Ethics Committee of Akita University (approval numbers: a-1-0412 and b-1-0408), as previously described [23]. To induce pathological cardiac hypertrophy, mice received daily intraperitoneal injections of ISO (30 mg/kg/day) for 14 days [24]. In parallel, PG extract (50 mg/kg/day) was administered by oral gavage in 0.5% (w/v) CMC in distilled water. ISO and PG (or vehicle) were administered at the same time each day to ensure consistent exposure. Control animals received vehicle alone at equivalent volumes (10 µL/g body weight). Animals were allocated to each treatment group using simple random assignment to minimize potential selection bias. Each experimental group consisted of four to five mice, and all animals survived the entire treatment period without meeting exclusion criteria. The dose of PG was selected based on previous reports demonstrating cardioprotective efficacy [23] and adjusted to approximate in vitro effective concentrations, considering reported oral bioavailability. Cardiac function was evaluated by transthoracic echocardiography using the Vevo 770 system (FUJIFILM VisualSonics Inc., Toronto, Canada) under ketamine (50 mg/kg) and xylazine (5 mg/kg) anesthesia. Fractional shortening (FS%), ejection fraction (EF%), left ventricular end-systolic diameter (LVESD), and end-diastolic diameter (LVEDD) were calculated from M-mode tracings. After treatment, mice were euthanized by cervical dislocation, and hearts were excised for histological analysis. Heart weight-to-body weight (HW/BW) ratios were determined. Paraffin-embedded sections were stained with hematoxylin-eosin (HE) and Masson’s trichrome (MT) to evaluate myocardial hypertrophy and fibrosis. Images were acquired using a BZ-X800 inverted microscope (KEYENCE, Tokyo, Japan) according to the protocol described previously [23]. All echocardiographic analyses and histological quantifications were performed by investigators blinded to group allocation.

Primary cardiomyocyte culture and hypertrophy induction

Neonatal Wistar rats (postnatal day 1-3; Japan SLC, Inc., Shizuoka, Japan) were used for NRVM isolation using the Pierce Cardiomyocyte Isolation Kit (Thermo Fisher Scientific, Waltham, MA, USA). Cells were cultured in Dulbecco’s modified Eagle’s medium (DMEM) supplemented with 10% fetal bovine serum, followed by serum-free conditions before stimulation. Hypertrophy was induced with ET-1 (100 nM, 48 h). ET-1, originally identified as a potent vasoconstrictor produced by vascular endothelial cells [25], exerts a range of cardiovascular effects, including the induction of cardiomyocyte hypertrophy [26-28]. ET-1 is widely used to model pathological cardiac hypertrophy at a concentration of 100 nM in previous studies [28-31]. Therefore, we employed primary NRVMs and treated them with 100 nM ET-1 to investigate hypertrophic remodeling and cytotoxicity. Quantification was performed using ImageJ software (National Institutes of Health, Bethesda, MD, USA), as previously reported [23]. Pharmacological intervention with MBT or crude drug extracts (PG, SA, CC, GP) was applied at concentrations corresponding to their respective contents within MBT.

Time-course to define endpoints. Before pharmacological testing, a pilot time-course was performed in NRVMs treated with ET-1 (100 nM) for 0, 12, 24, 48, and 72 h to determine the optimal analysis windows. Cell cross-sectional area (CSA) progressively increased (1.4 ± 0.1-fold at 12 h, 1.7 ± 0.1-fold at 24 h, and 2.1 ± 0.1-fold at 48 h vs. control), followed by a marked decline in viability at 72 h. Based on these kinetics, subsequent assays used 48 h for hypertrophy/RT-qPCR and 72 h for viability and cytotoxicity measurements.

Mitochondrial and cellular functional assays

Mitochondrial morphology and membrane potential were assessed using MitoTracker (Thermo Fisher Scientific) and confocal microscopy. Mitochondrial length was quantified via Gaussian fitting using ImageJ and Clampfit software (Molecular Devices, San Jose, CA, USA) [23]. Intracellular Ca^2+^ and ROS levels were measured using Fluo-4 AM and H_2_DCFDA, respectively. NRVMs were stimulated with 100 nM ET-1 for 48 h before measurements. Cells were incubated with 5 μM Fluo-4 AM or 5 μM H_2_DCFDA in Hank’s balanced salt solution (HBSS) for 30 min at 37°C. After washing, fluorescence was measured at excitation/emission = 485/538 nm using a microplate reader. All imaging and fluorescence-detection instruments were calibrated according to the manufacturer’s standard procedures before each experimental session to ensure measurement reliability. Thus, Ca²⁺ and ROS levels represent the status 48 h after ET-1 stimulation. ATP content was quantified with a luciferase-based kit (Fujifilm Wako Pure Chemical Corporation, Osaka, Japan). The experimental procedures followed those described previously [23].

Cell viability and cytotoxicity assays

NRVM viability was evaluated using the Cell Counting Kit-8 (CCK-8; Dojindo Laboratories, Kumamoto, Japan), while cytotoxicity was assessed by measuring lactate dehydrogenase (LDH) release (LDH Assay for Cytotoxicity; Dojindo Laboratories). Absorbance was measured at 450 nm (CCK-8) and 490 nm (LDH). Measurements were taken 72 h after ET-1 or crude medicine exposure [23]. For LDH cytotoxicity, absorbance from lysed cells (maximum LDH release) was defined as 100%, and absorbance from untreated control cells was defined as 0%. The cytotoxicity of each sample was calculated as a percentage of this dynamic range.

Gene and protein expression analysis

Total RNA was extracted from NRVMs using the NucleoSpin RNA Plus Kit (Takara Bio Inc., Shiga, Japan) and reverse transcribed with PrimeScript II (Takara Bio Inc.). Quantitative real-time polymerase chain reaction (qRT-PCR) was performed using SYBR Green reagents (Toyobo Co., Ltd., Osaka, Japan) and the LightCycler 480 system (Roche Diagnostics, Basel, Switzerland). The rat primer sequences used were as follows: ANP (NM_012612.2, 105 bp): F 5’-AAATCCCGTATACAGTGCGG-3’; R 5’-GGAGGCATGACCTCATCTTC-3’BNP (NM_031545.1, 364 bp): F 5’-CCATCGCAGCTGCCTGGCCCATCACTTCTG-3’; R 5’-GACTGCGCCGATCCGGTC-3’ Opa1 (NM_133585.3, 189 bp): F 5’-AAGAACCTGGAATCTCGAGGAGTCG-3’; R 5’-CCAGAACAGGACCACGTCGTTGC-3’ Mfn1 (NM_138976.2, 140 bp): F 5’-CTCGGAATCAACGCTGATGAAC-3’; R 5’-TGCGCACATCCTCCATATATTCT-3’ Mfn2 (NM_130894.4, 412 bp): F 5’-CTCAGGAGCAGCGGGTTTATTGTCT-3’; R 5’-TGTCGAGGGACCAGCATGTCTATCT-3’ Fis1 (NM_001401051.1, 130 bp): F 5’-ACAATGACGACATCCGTAGAGG-3’; R 5’-GCCTTTTCATATTCCTTGAGCCG-3’ Drp1 (NM_053655.3, 148 bp): F 5’-AGAATATTCAAGACAGCGTCCCAAAG-3’; R 5’-CGCTGTGCCATGTCCTCGGATTC-3’ Mtfp1 (NM_001006960.1, 102 bp): F 5’-AGATGAAGGCCCTGAGGAGT-3’; R 5’-CCAGTGTTCGTTCCCACTCA-3’ β-actin (NM_031144.3, 77 bp): F 5’-ACTATCGGCAATGAGCGGTTC-3’; R 5’-ATGCCACAGGATTCCATACCC-3’. Gene expression levels were normalized to β-actin and quantified using the ΔΔCt method. Primer sequences and PCR conditions were based on previously reported protocols [23].

Statistical analysis

Data are presented as mean ± standard error of the mean (SEM). Statistical significance was determined by Student’s t-test or one-way analysis of variance (ANOVA) with Tukey’s post hoc test. Analyses were conducted using GraphPad Prism 9 (GraphPad Software Inc., San Diego, CA, USA), with P < 0.05 considered significant. Each experiment was performed in at least three independent biological replicates. Data normality was assessed using the Shapiro-Wilk test, and homogeneity of variance was evaluated using Levene’s test. Parametric tests (t-test or ANOVA) were applied only when these assumptions were satisfied; Welch’s correction was used when variances were unequal.

## Results

PG exerts dose-dependent pharmacological efficacy against ET-1-induced hypertrophy in NRVMs

To model pathological hypertrophy, NRVMs were stimulated with 100 nM ET-1 following established protocols [28-31]. A preliminary time-course established 48 h as the optimal window for assessing hypertrophy and 72 h for viability/cytotoxicity (see Appendix A). Accordingly, we assessed hypertrophy at 48 h and viability/cytotoxicity at 72 h in subsequent experiments.

**Figure 1:**
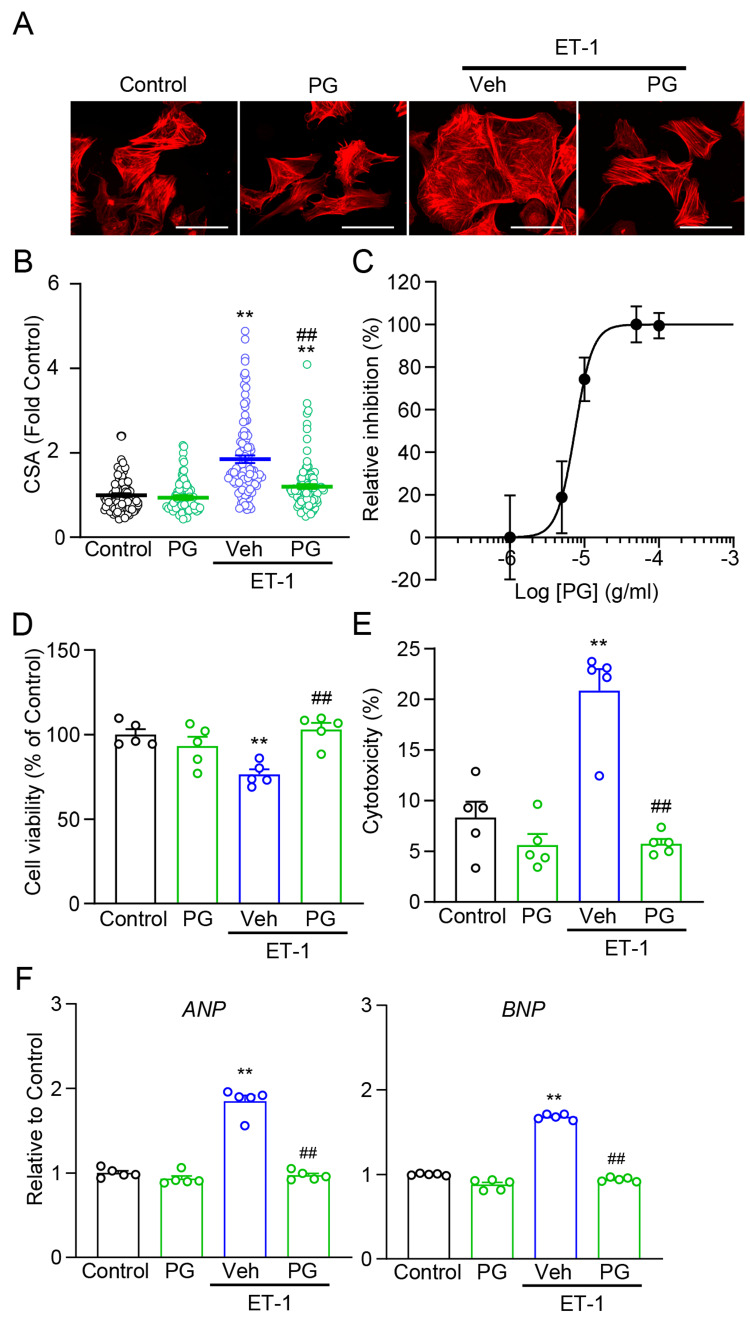
PG attenuates ET-1-induced hypertrophy with concentration-response and preserves cell viability in NRVMs NRVMs were stimulated with ET-1 (100 nM); hypertrophy endpoints were assessed at 48 h and viability/cytotoxicity at 72 h; vehicle groups received DMSO at matched final concentrations. (A) Representative phalloidin-stained images of Control, PG, ET-1 + Vehicle (Veh), and ET-1 + PG. Scale bar, 50 μm. (B) Quantification of cell cross-sectional area (CSA; fold Control) across groups (n = 101-113 cells/group). (C) Dose-response inhibition curve of PG against ET-1–induced CSA enlargement (IC_50_ = 7.5 μg/mL; Hill slope = 3.6; n = 105-115 cells/group). (D) Cell viability at 72 h (n = 5). (E) Cytotoxicity at 72 h (n = 5). (F) qRT-PCR for ANP and BNP mRNA expression (relative to Control) at 48 h (n = 5). ^**^P < 0.01 vs. Control; ^##^P < 0.01 vs. ET-1 + Veh. "n" indicates biological replicates unless otherwise noted. PG: *Panax ginseng*; ET-1: endothelin-1; NRVMs: neonatal rat ventricular myocytes; DMSO: dimethyl sulfoxide; IC_50_: half maximal inhibitory concentration; qRT-PCR: quantitative real-time polymerase chain reaction; ANP: atrial natriuretic peptide; BNP: brain natriuretic peptide.

We then evaluated the pharmacological actions of PG in 100 nM ET-1-stimulated NRVMs. As shown in Figure 1, PG attenuated ET-1-induced hypertrophic remodeling in a dose-dependent manner, as evidenced by a reduction in cell CSA (Figures 1A-1C). A log-scaled concentration-response curve showed a clear inhibitory profile of PG on ET-1-induced CSA enlargement, with an IC_50_ of 7.5 µg/mL and a Hill slope of 3.6 (Figure 1C). Concomitantly, PG preserved cell viability, limited cytotoxicity, and downregulated fetal gene markers (atrial natriuretic peptide (ANP) and brain natriuretic peptide (BNP)) under ET-1 stress (Figures 1D-1F).

Among the four crude drugs composing MBT, PG exhibited the most robust and consistent antihypertrophic activity, whereas SA showed modest effects, GP had little to no activity, and CC increased cytotoxicity under the tested conditions (Figures 1A-1B, 1D-1F; see Appendix B). Collectively, these findings identify PG as the principal bioactive constituent within MBT, supported by ANP/BNP responses under ET-1 stress and comparative data for the parent formula (Appendix C). To directly compare the efficacy of the hybrid Kampo formulation MBT with its principal component PG, inhibition rates relative to ET-1 stimulation were calculated. MBT suppressed ET-1-induced hypertrophy by 64.7 ± 6.2%, while PG achieved 56.7 ± 7.9% inhibition (Δ = -2.6%, 95% CI -9.4 to 4.2, p = 0.43, paired t-test). These results indicate no statistically significant difference between MBT and PG at equivalent doses, suggesting comparable pharmacological efficacy and supporting the interpretation that PG accounts for most of MBT’s antihypertrophic activity under the present conditions.

PG improves cardiac function and limits pathological remodeling in an ISO-induced mouse model

**Figure 2:**
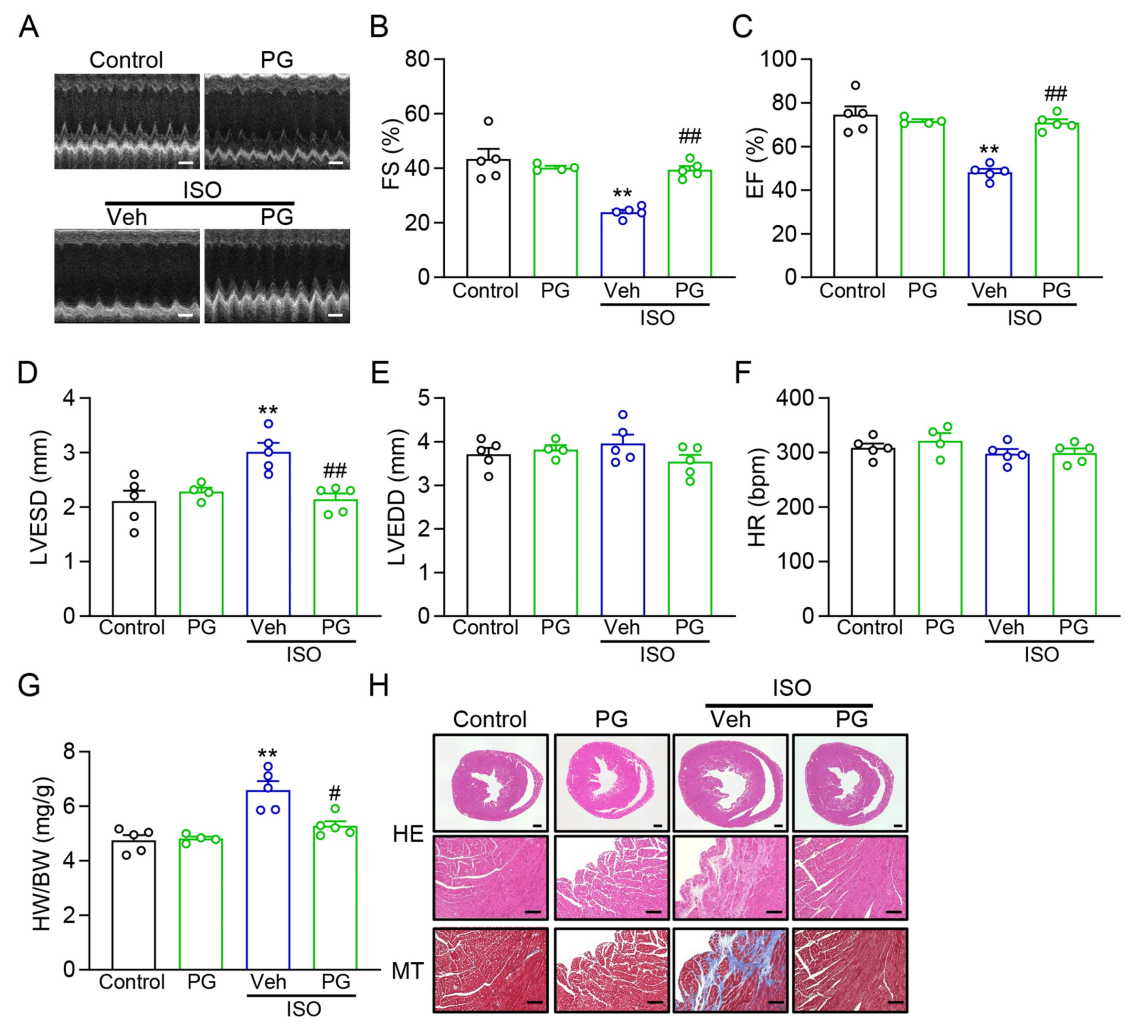
PG improves cardiac function and suppresses ISO-induced remodeling in vivo (A) Representative M-mode echocardiograms of mice with or without ISO and/or PG treatments. Scale bar, 200 ms. (B-F) Changes in the percentage of left ventricular (LV) fractional shortening (FS%) (B), LV ejection fraction (EF%) (C), LV end-systolic diameter (LVESD) (D), LV end-diastolic diameter (LVEDD) (E), and heart rate (HR) (F) (n = 4-5 mice). (G) Heart weight/body weight (HW/BW) ratio (n = 4-5 mice). (H) Histological analysis of heart tissue (HE and Masson staining). Scale bar, 500 µm (whole heart) and 100 µm (High magnification), respectively. ^**^P < 0.01 vs. Control; ^#^P < 0.05, ^##^P < 0.01 vs. ISO + Veh. PG: *Panax ginseng*; ISO: isoproterenol; HE: haematoxylin and eosin

We next assessed the in vivo pharmacological efficacy of PG in a mouse model of ISO-induced cardiac stress. To evaluate the in vivo cardioprotective effects of PG, we employed a mouse model of ISO-induced cardiac hypertrophy and dysfunction. PG was administered orally at 50 mg/kg/day, based on previous reports and the effective in vitro concentrations identified in this study [32]. Daily ISO (30 mg/kg, i.p., 14 days) impaired systolic performance, as indicated by reduced fractional shortening (FS%) and EF%, together with increased LV end-systolic diameter (LVESD) on M-mode echocardiography (Figures 2A-2D). Oral PG (50 mg/kg/day) significantly ameliorated these ISO-induced functional deficits, restoring FS% and EF% toward control levels and reducing LVESD. LV end-diastolic diameter (LVEDD) was not significantly altered across groups (Figure 2E), and heart rate (HR) remained comparable, excluding chronotropic confounding (Figure 2F).

Consistent with the functional rescue, PG attenuated hypertrophic remodeling, evidenced by a lower heart weight-to-body weight (HW/BW) ratio relative to ISO alone (Figure 2G). Histological analysis further revealed that ISO induced interstitial fibrosis and collagen deposition, which were markedly reduced by PG treatment, as indicated by HE and MT staining (Figure 2H).

Together, these data demonstrate that PG confers pharmacological cardioprotection in vivo, improving systolic function and limiting structural remodeling in the ISO model without affecting baseline HR or LV filling (LVEDD).

PG preserves mitochondrial morphology and membrane potential under ET-1 stress

**Figure 3:**
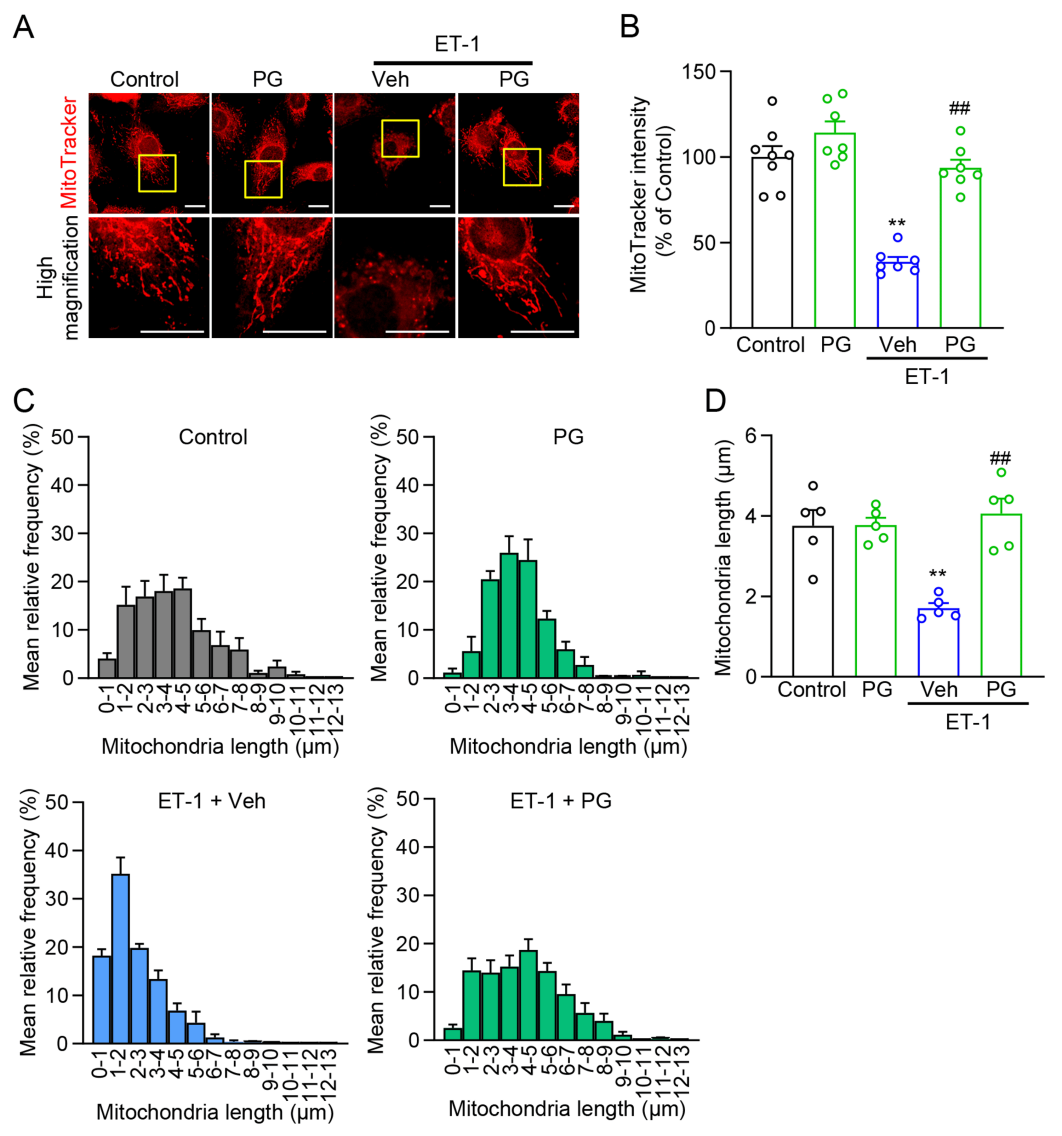
PG preserves mitochondrial integrity under ET-1 stress in NRVMs (A) Representative MitoTracker images under Control, PG, ET-1 + Vehicle (Veh), and ET-1 co-treatment with PG. Scale bar, 20 μm. (B) Quantification of normalized MitoTracker fluorescence intensity (n = 7-8 fields/group). (C) Representative mitochondrial length estimates derived by Gaussian fitting of length distributions (n = 5 fields/group). (D) Mean mitochondrial length in each group in (C). ^**^P < 0.01 vs. Control; ^##^P < 0.01 vs. ET-1 + Veh. PG: *Panax ginseng*; ET-1: endothelin-1; NRVMs: neonatal rat ventricular myocytes

To determine whether the antihypertrophic actions of PG are accompanied by preservation of organellar integrity, we examined mitochondrial structure and membrane potential in ET-1-stimulated NRVMs using MitoTracker imaging. ET-1 (100 nM, 48 h) induced a fragmented mitochondrial network, accompanied by a reduction in MitoTracker signal intensity, consistent with a loss of membrane potential. PG attenuated ET-1-induced mitochondrial fragmentation-maintaining a more reticular/elongated network-and mitigated the ET-1-induced loss of MitoTracker signal toward control levels (Figures 3A-3B). Quantitatively, ET-1 shifted mitochondrial length distributions toward shorter values, whereas PG significantly increased the fitted mean/median length relative to ET-1 + vehicle, indicating attenuation of fragmentation (Figure 3C). In parallel, PG attenuated the ET-1-induced decrease in normalized MitoTracker fluorescence compared with ET-1 alone (Figure 3B). In head-to-head comparisons with other MBT constituents evaluated at matched, MBT-equivalent concentrations, SA showed only limited mitigation, GP showed little to no effect, and CC did not ameliorate (and in some fields exacerbated) the ET-1-induced morphological changes (see Appendices D, E).

Additional exemplar fields for MitoTracker imaging are shown in Appendix D, full mitochondrial length distributions for PG and comparators are in Appendix E, and MBT-focused extensions are presented in Appendices F-G.

PG mitigates cellular stress and supports mitochondrial function

**Figure 4:**
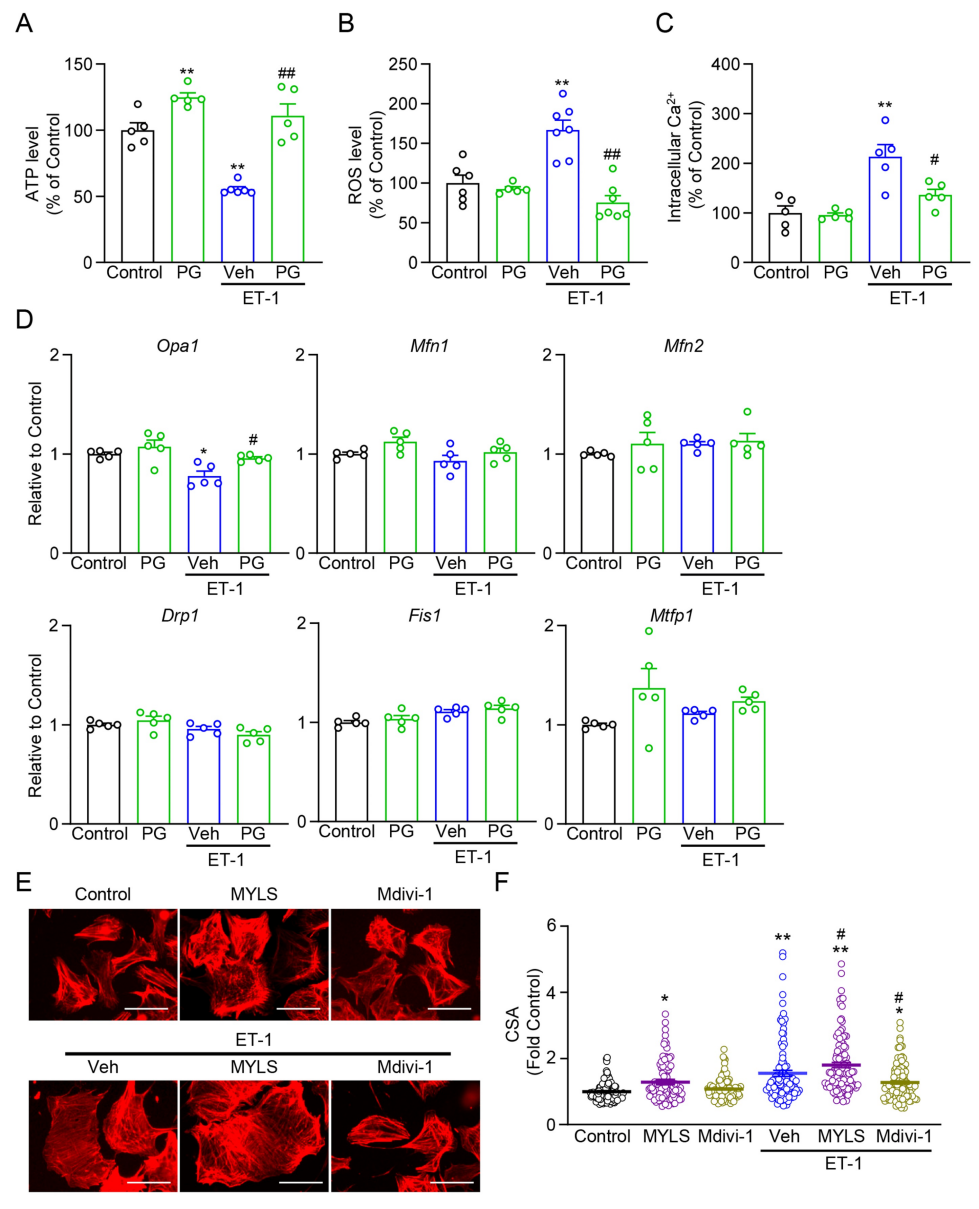
PG modulates mitochondrial fission/fusion gene expression and cellular stress markers (A-C) Quantification in NRVMs of ATP content (A), ROS (H_2_DCFDA fluorescence) (B), and cytosolic Ca^2+^ (Fluo-4 fluorescence) (C), expressed as % of Control (n = 5-7 per panel). (D) qRT-PCR analysis of mitochondrial fusion (Opa1, Mfn1, Mfn2) / fission (Drp1, Fis1, Mtfp1) genes at 48 h (n = 5). (E) Representative phalloidin-stained images of Control, MYLS22 (Opa1 inhibitor), Mdivi-1 (Drp1 inhibitor), ET-1 + Vehicle (Veh), ET-1 + MYLS22, and ET-1 + Mdivi-1. Scale bar, 50 μm. (F) Cell CSA under ET-1 with pharmacological modulation: MYLS22 and Mdivi-1 (n = 104-117 cells/group). ^*^P < 0.05, ^**^P < 0.01 vs. Control; ^#^P < 0.05, ^##^P < 0.01 vs. ET-1 + Veh. "n" indicates biological replicates unless otherwise noted. PG: *Panax ginseng*; NRVMs: neonatal rat ventricular myocytes; ATP: adenosine triphosphate; ROS: reactive oxygen species; qRT-PCR: quantitative real-time polymerase chain reaction; ET-1: endothelin-1; Veh: vehicle; CSA: cross-sectional area

To complement the morphological readouts, we next quantified indices of cellular stress and mitochondrial fusion/fission signaling. In ET-1-stimulated NRVMs, ATP content was reduced, while ROS and intracellular Ca^2+^ were elevated versus control. PG treatment significantly increased ATP and lowered ROS and Ca^2+^ toward baseline (Figures 4A-4C), consistent with a broad attenuation of stress burden under hypertrophic conditions (Figures 1-3).

To elucidate the molecular basis of these effects, we analyzed the expression of genes involved in mitochondrial fusion (Opa1, Mfn1, Mfn2) and fission (Drp1, Fis1, Mtfp1). ET-1 selectively downregulated Opa1, whereas Mfn1/Mfn2/Drp1/Fis1/Mtfp1 showed no consistent changes. PG restored Opa1 expression to control levels without materially altering the expression of other fusion/fission transcripts (Figure 4D), supporting a fusion-preserving mechanism rather than a global shift in mitochondrial dynamics gene expression.

To functionally contextualize these observations, we employed pharmacological modulators: MYLS22 (an Opa1 inhibitor) and Mdivi-1 (a Drp1 inhibitor that suppresses mitochondrial fission). Opa1 inhibition with MYLS22 increased CSA and exacerbated ET-1-induced hypertrophy, whereas Drp1 inhibition with Mdivi-1 tempered CSA enlargement in the presence of ET-1 (Figure 4E). Together with the transcriptional data, these interventions indicate that maintaining fusion capacity (Opa1) and/or restraining fission (Drp1) counteract hypertrophic remodeling, and that PG aligns with the fusion-preserving axis under ET-1 stress. It should be noted that the current evidence for Opa1 involvement is based on transcript-level changes and phenotypic readouts. Although protein-level confirmation (e.g., Western blot, immunostaining) would further substantiate this link, our goal here was to delineate a mitochondria-preserving pharmacological phenotype rather than to establish direct protein-level causality. Complementary data for the parent formula MBT under ET-1 stress, including mitochondrial morphology, membrane potential, and cellular homeostasis (ATP/ROS/Ca^2+^), are provided in Appendix F, with quantitative summaries of mitochondrial length distributions in Appendix G.

## Discussion

The present study demonstrates that PG, a major bioactive constituent of the Kampo formula MBT, exerts pharmacological cardioprotection in two experimental systems: a cellular model of hypertrophy using ET-1-stimulated NRVMs and an animal model of cardiac stress using ISO-treated mice. PG suppressed ET-1-evoked hypertrophic remodeling in NRVMs with a clear concentration-response (IC_50_ = 7.5 µg/mL) and improved systolic function while limiting structural remodeling in an ISO-induced mouse model (Figures 1-2). At the cellular level, PG preserved mitochondrial organization and membrane potential, while improving ATP production and reducing ROS and cytosolic Ca^2+^ levels (Figures 3-4), supporting a mitochondria-preserving mode of action.

ET-1 stimulation is known to induce mitochondrial fragmentation, ROS accumulation, Ca^2+^ overload, and ATP depletion, key contributors to cardiomyocyte hypertrophy and injury [33-36]. Although the in vivo model employs ISO rather than ET-1, both stimuli converge on a common downstream axis of Ca^2+^ overload, mitochondrial dysfunction, and oxidative stress. This mechanistic overlap provides the bridge between our cell and animal data and justifies evaluating PG across both paradigms. Our NRVM data recapitulate these stress signatures under ET-1 and show that PG counters each component, maintaining mitochondrial network integrity and membrane potential (Figure 3), restoring ATP, and dampening ROS and Ca^2+^ rises (Figure 4). These effects are consistent with previous reports on the mitochondrial-modulating actions of ginsenosides, such as Rc, Rg1, and Rd, which activate mitophagy and promote mitochondrial biogenesis via SIRT1/PINK1/Parkin and omentin-1 signaling, respectively [37-41]. Our finding that PG reduces ROS (Figure 4B) is consistent with the known antioxidant properties of PG-containing formulations in cardiovascular disease models [42]. Notably, PG extract has also been reported to regulate mitochondrial function and suppress calcineurin activity [43,44]. Although both ET-1 and ISO ultimately elicit hypertrophic stress, they act through distinct upstream signaling-endothelin-receptor versus β-adrenergic-and differ in temporal and systemic context. This dual approach provides complementary insight into how PG preserves mitochondrial integrity across acute and chronic neurohumoral stress conditions. Given that both ET-1 and ISO converge on mitochondrial and Ca^2+^ dysregulation, it is important to consider the early-stage pathophysiological events shared across these models. Early hypertrophic remodeling induced by ET-1 is driven by Ca²⁺ overload and mitochondrial dysfunction [29-32], and our NRVM data at 48 h reproduce these early pathogenic signatures. Importantly, similar early impairments in Ca^2+^ handling and mitochondrial homeostasis have also been documented in ISO-induced cardiac remodeling. ISO stimulation activates CaMKIIδ-dependent RyR2 phosphorylation, enhancing SR Ca^2+^ leak during the early phase of β-adrenergic stress [45]. Furthermore, Ca^2+^-dependent mitochondrial injury has been shown to precipitate cardiomyocyte necrosis and functional decline in ISO-stimulated hearts [46]. In zebrafish models, ISO rapidly reduces Ca^2+^ transients and contractility before the later collapse of mitochondrial ATP production [47]. These findings collectively suggest that PG may counteract early Ca^2+^ and mitochondrial disturbances during the initial phase of ISO-induced cardiac hypertrophy.

In vivo analysis confirmed the functional relevance of these cellular effects. In ISO-treated mice, PG restored systolic performance, improving FS% and EF% and reducing LVESD, without altering HR, and LVEDD remained unchanged (Figures 2A-2F), arguing against chronotropic or preload confounders. PG also reduced HW/BW and attenuated interstitial fibrosis (Figures 2G-2H). These outcomes are consistent with prior reports that PG or ginsenoside derivatives reverse established hypertrophy and improve function after myocardial injury or HF [44,48], thereby validating a cardioprotective profile under pathological stress in an independent β-adrenergic model.

Contrastingly, the CC, another component of MBT, exhibited cytotoxic effects on NRVMs (Appendices B, D, E). While trans-cinnamaldehyde, the main active compound in CC, has been shown to alleviate cardiac hypertrophy and fibrosis through ERK inhibition, CaMKII modulation, and correction of Ca^2+^ handling [49], our findings raise concerns about the cardiotoxicity of its DMSO-soluble fraction. This discrepancy underscores the importance of isolating individual components and testing them under defined experimental conditions. Such cytotoxicity may result from concentration-dependent effects or altered solubility and bioavailability, highlighting the need for refined pharmacological evaluation of crude drug constituents.

Mechanistically, our data are consistent with a fusion-preserving pharmacological phenotype and *Opa1*-associated transcriptional restoration; however, causality was not established in the present study. Although our data demonstrate transcriptional restoration of Opa1 and phenotypic alignment with fusion-preserving pharmacology, these observations remain correlative. We did not perform molecular docking, target-binding, or genetic manipulation of Opa1 in the present study. Therefore, our findings should be interpreted as indicative of an Opa1-associated, rather than Opa1-mediated, mechanism. Future studies incorporating biochemical target engagement assays and cardiomyocyte-specific Opa1 gain- or loss-of-function approaches will be necessary to establish direct causality. Although our in vivo findings demonstrate significant improvements in systolic function and attenuation of fibrosis with PG treatment, the relatively small sample size (n = 4-5 per group) may limit the statistical power to detect more subtle physiological or structural differences. Future studies using larger cohorts and additional hypertrophy models will be essential to confirm and extend these observations. ET-1 selectively downregulated Opa1 in NRVMs, and PG restored Opa1 expression toward control without consistent shifts in Mfn1/Mfn2/Drp1/Fis1/Mtfp1 (Figure 4D). Pharmacological probing was concordant: MYLS22, a reversible, noncompetitive Opa1 GTPase inhibitor identified by high-throughput screening and shown to induce cristae remodeling/fragmentation by engaging Opa1 [50], exacerbated hypertrophy (increased CSA) under ET-1, whereas Mdivi-1, a Drp1 GTPase inhibitor that reduces fission and has been reported to decrease Drp1/Fis1 and increase Mfn1/Mfn2/Opa1 with improved mitochondrial function [51], attenuated CSA enlargement (Figure 4E). These observations align with independent evidence that Opa1 activity is cardioprotective: Opa1-induced mitophagy preserves cardiomyocyte viability after myocardial infarction and is activated by irisin, whereas Opa1 knockdown abolishes irisin’s benefits [52]; and the natural polyphenol punicalagin protects against diabetic cardiomyopathy by promoting Opa1-mediated mitochondrial fusion via a PTP1B-STAT3-Opa1 axis, with Opa1 knockdown abrogating protection [53]. Taken together, our transcriptional rescue (Figure 4D) and orthogonal pharmacology (Figure 4E), in the context of these reports, support the inference that maintaining Opa1-dependent fusion capacity (and/or restraining Drp1-dependent fission) counteracts hypertrophic remodeling and that PG aligns with the fusion-preserving axis, while we appropriately refrain from asserting strict causality pending genetic gain/loss-of-function validation.

Positioning PG within its parent formula, MBT also enabled us to identify the effective constituent of the mixture. In head-to-head assays at MBT-equivalent concentrations, PG produced the most consistent antihypertrophic (CSA, ANP/BNP) and cytoprotective effects. In contrast, SA was modest, GP was inactive, and CC increased cytotoxicity under some conditions (Appendix B). By analyzing PG alongside MBT, we were able to pinpoint PG as the bioactive driver within the formula, while still documenting the performance of MBT: antihypertrophic/viability readouts are summarized in Appendices B, C, and additional distribution-level comparisons for PG vs. SA/CC/GP are provided in Appendices D-G. Together with prior evidence that MBT mitigates cardiomyocyte hypertrophy in related settings [23], these findings indicate that, in the context of ET-1-induced injury, PG attenuated mitochondrial fragmentation and membrane potential loss, prevented ATP depletion, and reduced stress markers (ROS and Ca^2+^), consistent with a protective rather than baseline-enhancing effect.

Strengths of this work include (i) a dose-dependent in vitro pharmacology in ET-1-stimulated NRVMs on canonical hypertrophy readouts (CSA, ANP/BNP; IC_50_ = 7.5 µg/mL), (ii) in vivo efficacy on clinically relevant echocardiographic indices (FS%, EF%, LVESD) with no confounding changes in HR or LVEDD, and (iii) mechanistic coherence across ATP/ROS/Ca^2+^ and mitochondrial readouts that converge on Opa1-supported fusion. Limitations include reliance on transcript-level Opa1 changes and pharmacologic probes (MYLS22, Mdivi-1) rather than genetic gain/loss-of-function in cardiomyocytes; absence of pharmacokinetics/pharmacodynamics and tissue exposure data linking ginsenoside levels to effect size; and testing in a single stress paradigm (ISO) rather than pressure-overload or ischemia/reperfusion models. Future work should also include pharmacokinetic and bioavailability analyses to correlate systemic exposure with pharmacodynamic efficacy, alongside the following objectives: (i) bioassay-guided fractionation with HPLC/LC-MS to pinpoint the active ginsenoside(s)/fractions; (ii) causal validation of the fusion axis using cardiomyocyte-targeted Opa1/Drp1 gain- and loss-of-function plus live mitophagy/biogenesis reporters; (iii) high-resolution functional readouts, Seahorse respiration, ATP flux, and Ca^2+^ transients, integrated with super-resolution imaging of mitochondrial networks; and (iv) biochemical target engagement assays for Opa1 (e.g., in vitro GTPase activity, thermal-shift), to link chemical exposure to mechanism.

## Conclusions

This study demonstrates that PG exhibits dose-dependent anti-hypertrophic activity in ET-1-stimulated cardiomyocytes and delivers functional and structural benefits in vivo in ISO-stressed hearts. Across models, its pharmacological signature, preservation of mitochondrial organization and membrane potential, restoration of ATP, and attenuation of ROS and Ca^2+^ stress, with Opa1 support, is consistent with a mitochondria-preserving mode of action. By benchmarking MBT constituents head-to-head, we also identified PG as the bioactive driver within the formula. These findings position PG as a natural product candidate for targeting mitochondrial dysfunction in pathological cardiac remodeling and provide a mechanistic framework to guide fractionation, genetic validation of the fusion axis, and target engagement assays in future studies.

## Figures and Tables

**Figure 5:**
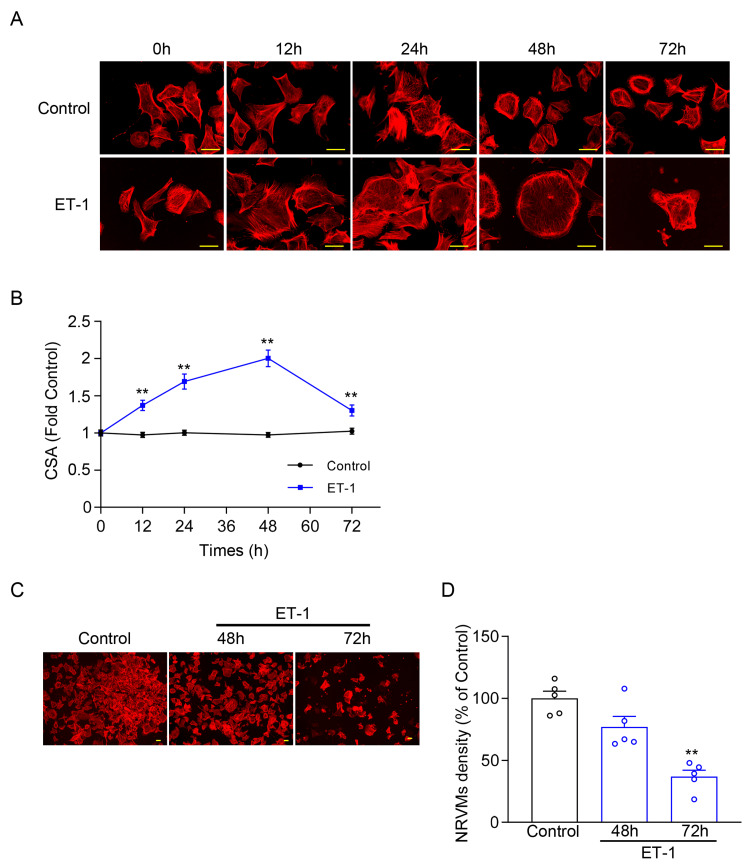
Time course analysis of ET-1-induced hypertrophic response and cell viability in NRVMs (A) Representative phalloidin-stained images at 0, 12, 24, 48, and 72 h after ET-1 (100 nM). Scale bar, 50 μm. (B) Quantification of cell cross-sectional area (CSA; fold Control) across time points (n = 102-114 cells/time point). (C) Rhodamine-conjugated phalloidin staining shows cell density at 48 and 72 h post ET-1 administration. Scale bar, 50 μm. (D) Quantification of cell density (n = 5 fields/group). These results justify 48 h for hypertrophy readouts and 72 h for viability/cytotoxicity assessments in the main figures. ^**^P < 0.01 vs. Control. ET-1: endothelin-1; NRVMs: neonatal rat ventricular myocytes

**Figure 6:**
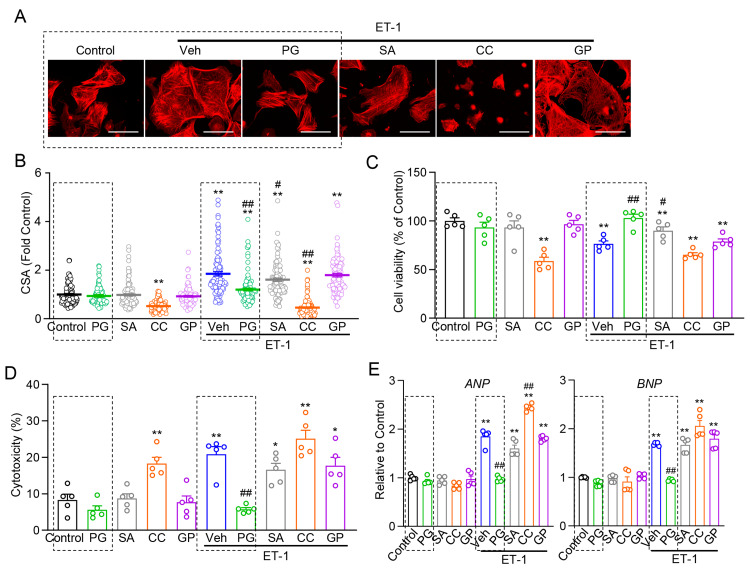
Constituent benchmarking identifies PG as the principal anti-hypertrophic component NRVMs were stimulated with ET-1 (100 nM) for 48 h (hypertrophy) or 72 h (viability/cytotoxicity). (A) Representative phalloidin-stained images under ET-1 + Vehicle and ET-1 co-treatment with PG (50 μg/mL), SA (50 μg/mL), CC (50 μg/mL), GP (100 μg/mL). Scale bar, 50 μm. (B) CSA quantification (n = 101–113 cells/group). (C) Cell viability at 72 h (n = 5). (D) Cytotoxicity at 72 h (LDH release; n = 4–5). (E) qRT-PCR for ANP and BNP at 48 h (n = 5). ^*^P < 0.05, ^**^P < 0.01 vs. Control; ^#^P < 0.05, ^##^P < 0.01 vs. ET-1 + Veh. Data enclosed by dotted lines correspond to those shown in Figure 1 of the main text. "n" indicates biological replicates unless otherwise noted. PG: *Panax ginseng*; NRVMs: neonatal rat ventricular myocytes; ET-1: endothelin-1; SA: *Sinomenium acutum *stem extract; CC: Cinnamomi cortex extract; GP: Gypsum Fibrosum; CSA: cross-sectional area; LDH: lactate dehydrogenase; qRT-PCR: quantitative real-time polymerase chain reaction; ANP: atrial natriuretic peptide; BNP: brain natriuretic peptide; Veh: vehicle

**Figure 7:**
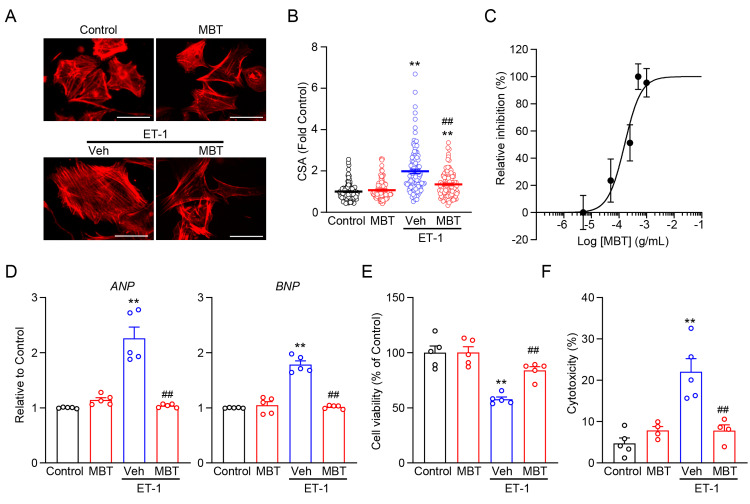
MBT attenuate ET-1-induced hypertrophic responses in NRVMs (A) Representative phalloidin-stained images under Control, MBT, ET-1 + Vehicle, and ET-1 + MBT (500 μg/mL). Scale bar, 50 μm. (B) CSA quantification (fold Control; n = 103-112 cells/group). (C) Dose-response inhibition curve of MBT against ET-1-induced CSA enlargement (IC_50_ of 159.2 μg/mL and a Hill slope of 1.1; n = 102-110 cells/group). (D) qRT-PCR of ANP/BNP at 48 h (n = 5). (E, F) Cell viability (E; n = 5) and cytotoxicity (F; n = 4-5) at 72 h. ^**^P < 0.01 vs. Control; ^##^P < 0.01 vs. ET-1 + Veh. "n" indicates biological replicates unless otherwise noted. MBT: Moku-boi-to; ET-1: endothelin-1; NRVMs: neonatal rat ventricular myocytes; CSA: cross-sectional area; IC_50_: half maximal inhibitory concentration; qRT-PCR: quantitative real-time polymerase chain reaction; ANP: atrial natriuretic peptide; BNP: brain natriuretic peptide; Veh: vehicle

**Figure 8:**
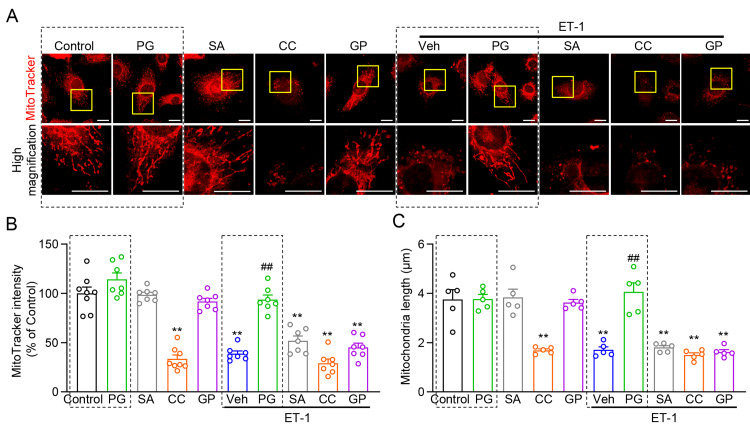
PG preserves mitochondrial integrity in ET-1-stimulated NRVMs (A) Representative MitoTracker images under Control, PG, SA, CC, GP, ET-1 + Vehicle, and each crude drug treatment (PG, SA, CC, GP); additional exemplar fields to complement main-figure quantifications. Scale bar, 20 μm. (B) Quantification of normalized MitoTracker fluorescence (n = 7-8 fields/group). (C) Mitochondrial length estimates derived by Gaussian fitting of length distributions (n = 5 per condition). ^**^P < 0.01 vs. Control; ^##^P < 0.01 vs. ET-1 + Veh. Data enclosed by dotted lines correspond to those shown in Figure 3 of the main text. PG: *Panax ginseng*; ET-1: endothelin-1; NRVMs: neonatal rat ventricular myocytes; SA: *Sinomenium acutum* stem extract; CC: Cinnamomi cortex extract; GP: Gypsum Fibrosum; Veh: vehicle

**Figure 9:**
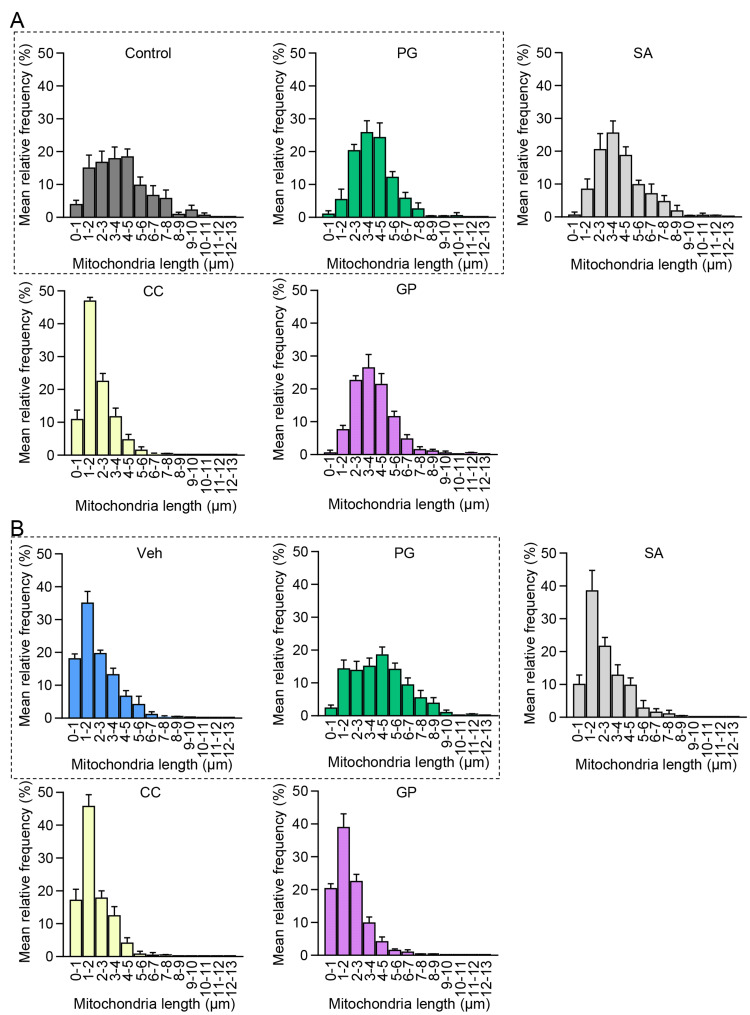
Distribution analysis of mitochondrial length in NRVMs treated under PG and comparator constituents (A) Control NRVMs treated with Vehicle, PG (50 μg/mL), SA (50 μg/mL), CC (50 μg/mL), or GP (100 μg/mL). (B) ET-1 (100 nM, 48 h) with the same treatments. Mitochondrial length distributions were compiled per cell and fit by Gaussian fitting (n = 5 per condition). Data enclosed by dotted lines correspond to those shown in Figure 3C of the main text. NRVMs: neonatal rat ventricular myocytes; PG: *Panax ginseng*; SA: *Sinomenium acutum* stem extract; CC: Cinnamomi cortex extract; GP: Gypsum Fibrosum; ET-1: endothelin-1

**Figure 10:**
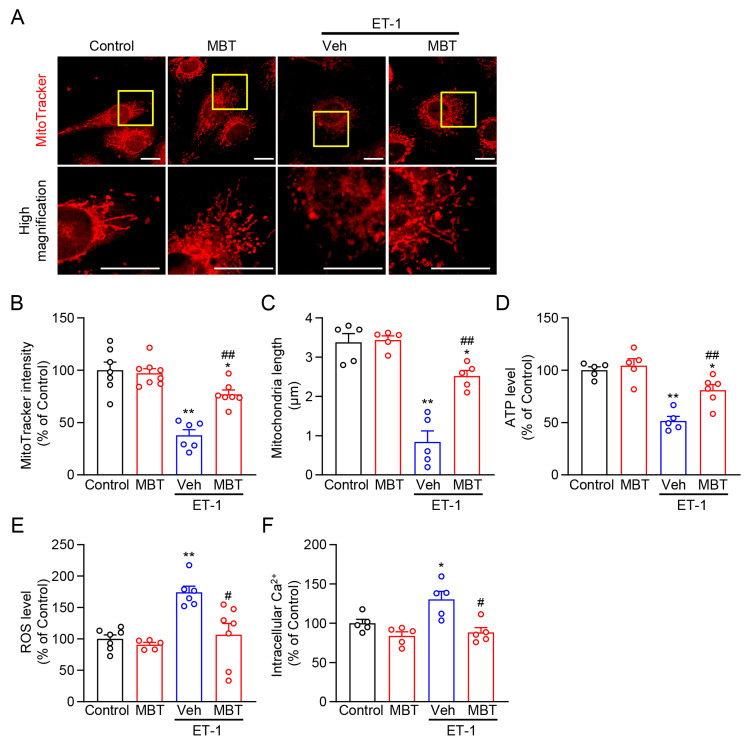
MBT preserves mitochondrial morphology and cellular homeostasis under ET-1 stress (A) Representative MitoTracker images under Control, MBT, ET-1 + Vehicle, and ET-1 + MBT (500 μg/mL). Scale bar, 20 μm. (B) Quantification of mitochondrial membrane potential (normalized MitoTracker fluorescence; n = 6–8 fields/group). (C) Mitochondrial length (Gaussian fitting; n = 5 per condition). (D-F) ATP (D; n = 5-6), ROS (E; n = 5-7), and cytosolic Ca^2+^ (F; n = 5). ^*^P < 0.05, ^**^P < 0.01 vs. Control. ^#^P < 0.05, ^##^P < 0.01 vs. ET-1 + Veh. "n" indicates biological replicates unless otherwise noted. MBT: Moku-boi-to; ET-1: endothelin-1; NRVMs: neonatal rat ventricular myocytes; Veh: vehicle; ATP: adenosine triphosphate; ROS: reactive oxygen species

**Figure 11:**
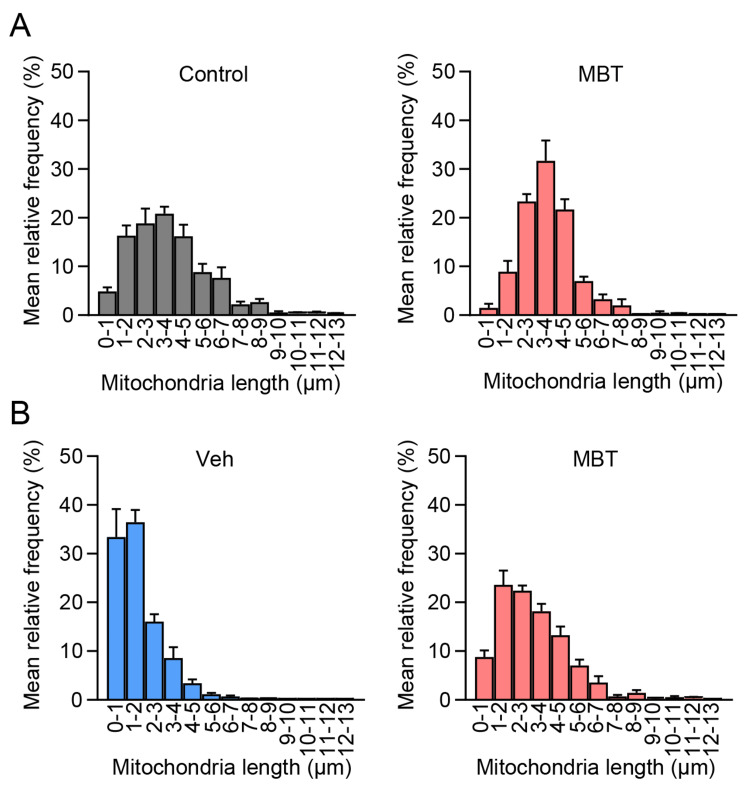
Quantitative analysis of mitochondrial morphology in NRVMs treated with ET-1 and MBT (A) Control NRVMs with Vehicle or MBT (500 μg/mL). (B) ET-1 (100 nM, 48 h) with Vehicle or MBT (500 μg/mL). Mitochondrial length distributions were analyzed as in Methods and summarized (n = 5 fields/group). NRVMs: neonatal rat ventricular myocytes; ET-1: endothelin-1; MBT: Moku-boi-to

## Appendices

Appendix A 

Appendix B 

Appendix C 

Appendix D 

Appendix E 

Appendix F 

Appendix G 
